# Supplementary material for: A Novel Anti-CD44 Variant 9 Monoclonal Antibody C44Mab-1 Was Developed for Immunohistochemical Analyses against Colorectal Cancers
Source: Curr Issues Mol Biol. 2023 Apr 20;45(4):3658–73. doi: 10.3390/cimb45040238 (PMC10137259; doi:10.3390/cimb45040238)
Supplement: Supplementary file 1 [file cimb-45-00238-s001.zip › cimb-2344447-supplementary Figure .pdf]

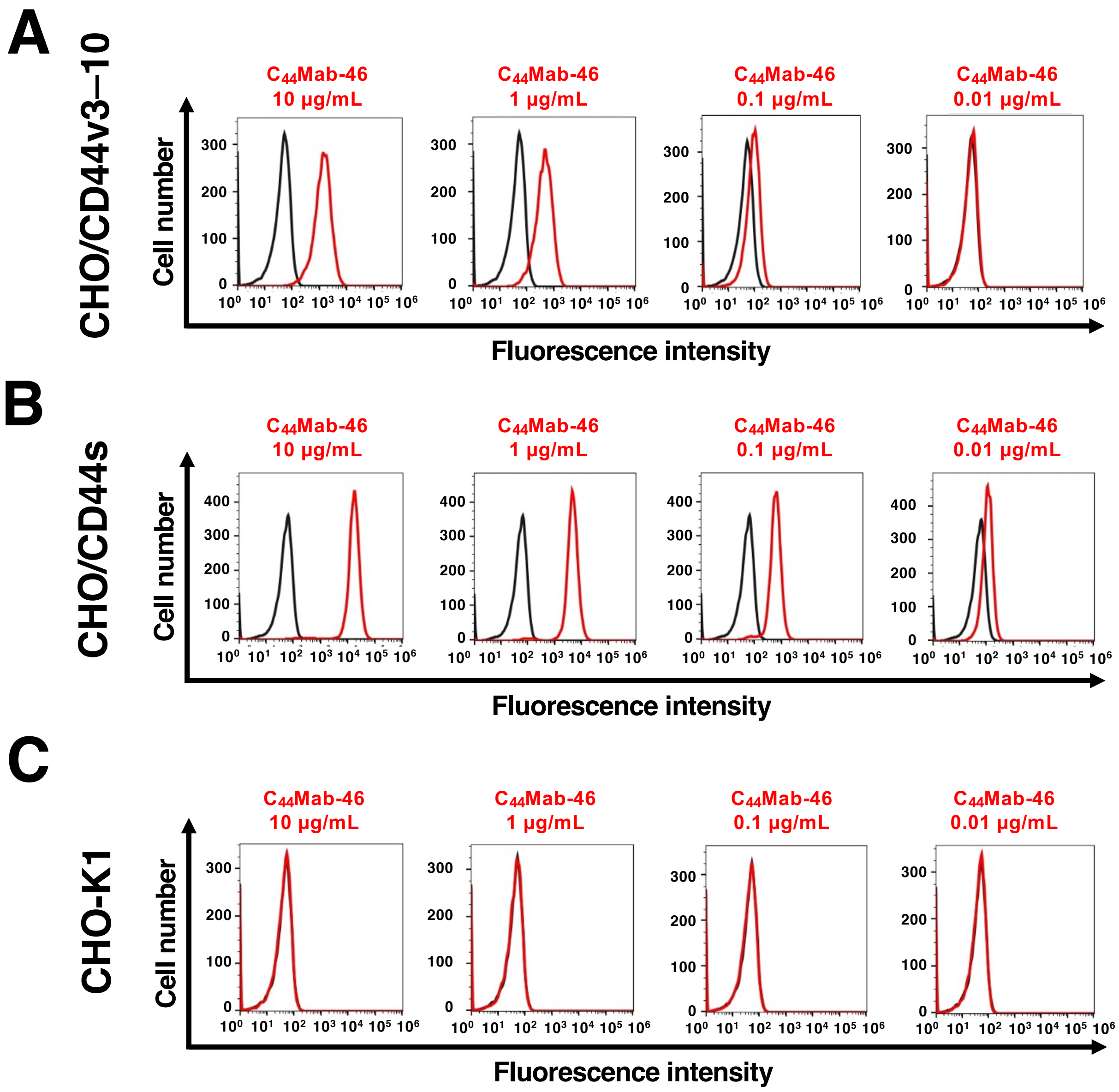

Figure S1 Conformation of the recognition of CHO/CD44s and CHO/CD44v3–10 by C_{44}Mab-46 by flow cytometry. CHO/CD44v3–10 (A), CHO/CD44s (B), and CHO-K1 (C) were treated with 0.01-10 μg/mL of C_{44}Mab-46, followed by treatment with Alexa Fluor 488-conjugated anti-mouse IgG (Red line). The black line represents the negative control (blocking buffer).

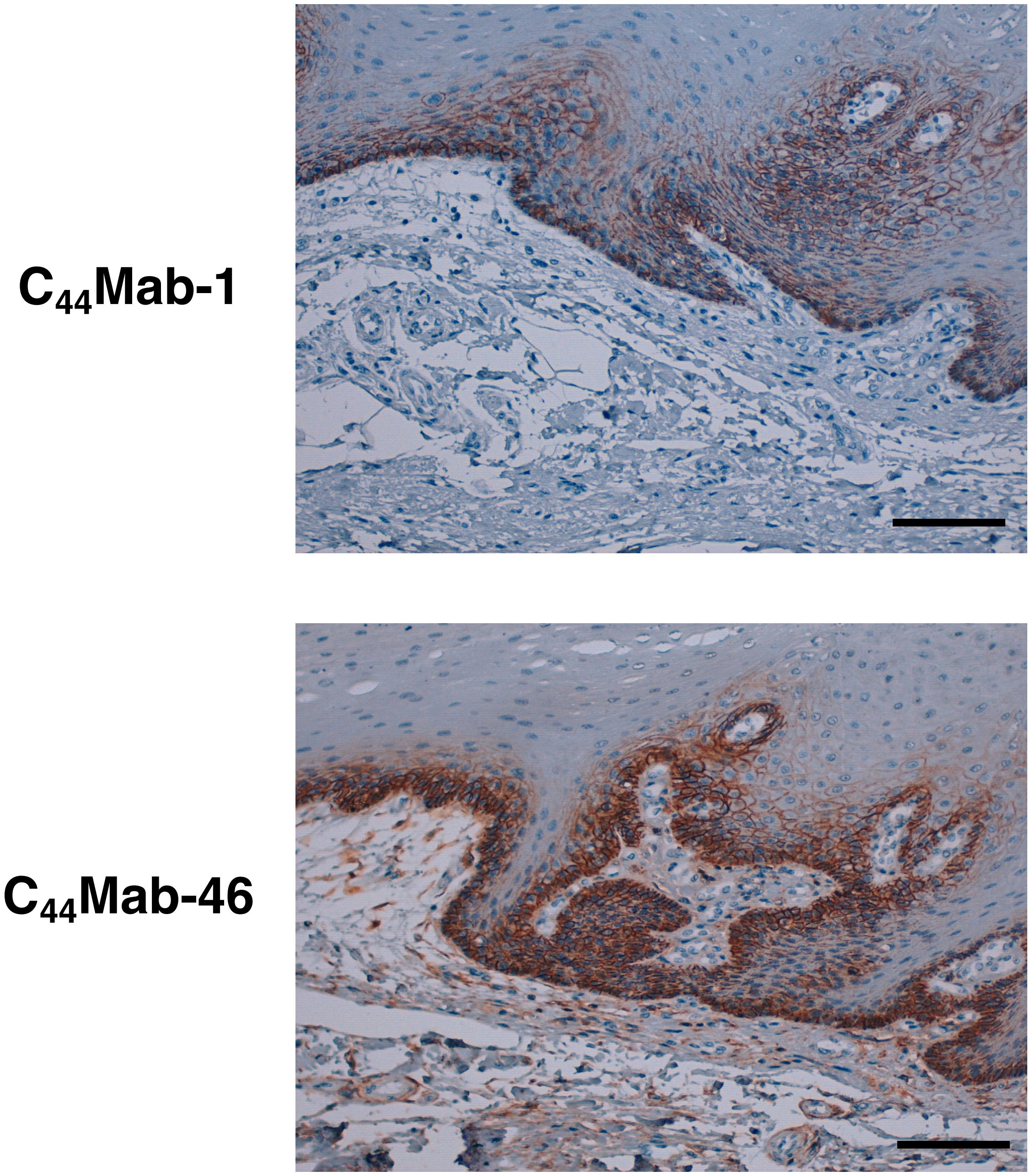

Figure S2 Immunohistochemical analysis using C_{44}Mab-1 and C_{44}Mab-46 against oral squamous cell carcinoma tissues. After antigen retrieval, the sections were incubated with 1 μg/mL of C_{44}Mab-1 (A) and 1 μg/mL of C_{44}Mab-46 (B), followed by treatment with the Envision+ kit. The color was developed using 3,3′-diaminobenzidine tetrahydrochloride (DAB), and the sections were counterstained with hematoxylin. Scale bar = 100 μm.

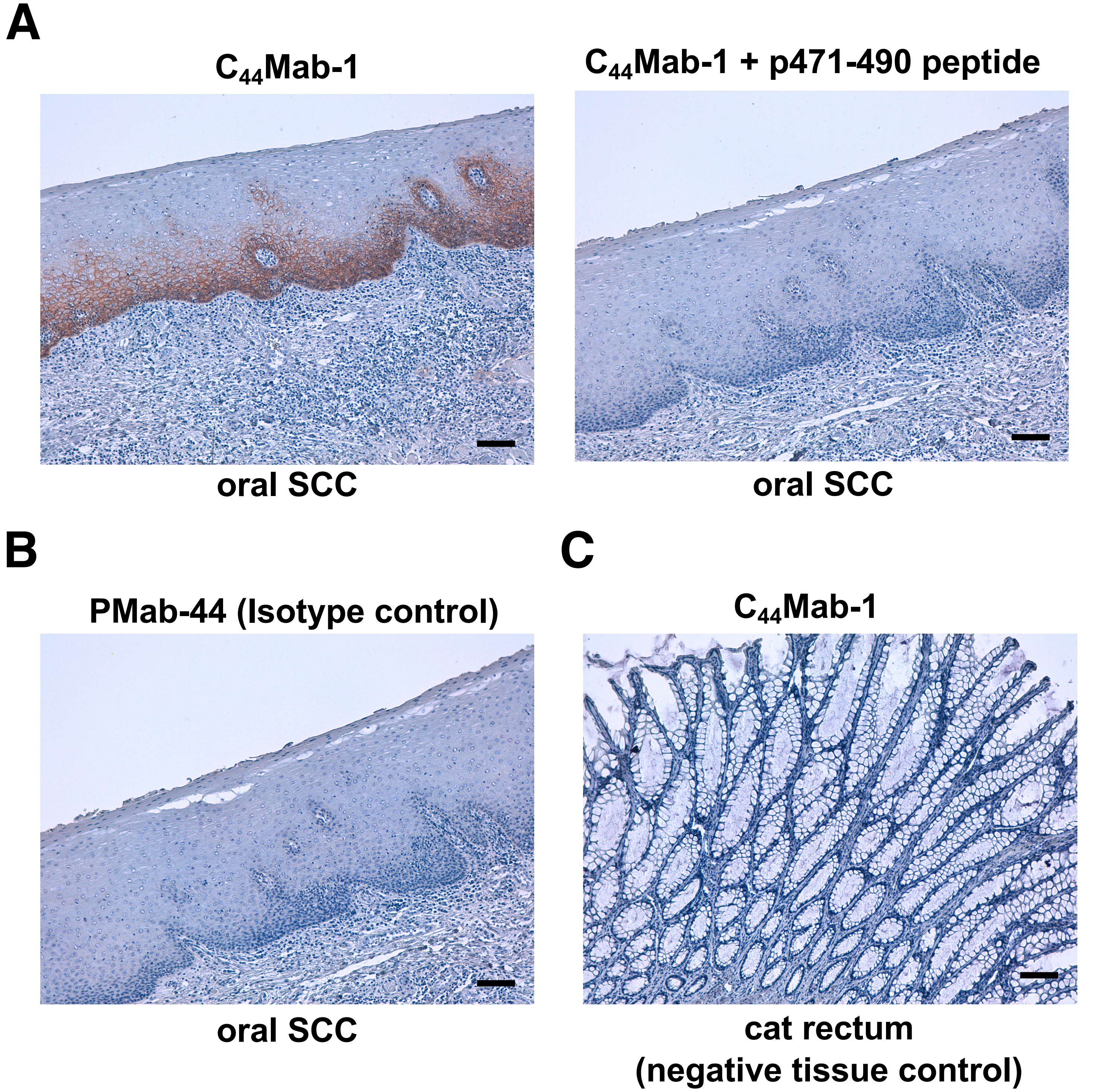

Figure S3 The blocking assay by an epitope peptide, isotype control, and negative tissue control in immunohistochemistry. (A) Blocking of the C_{44}Mab-1 reactivity to oral SCC tissue (positive tissue control) by the CD44 peptide (aa 471–490) containing the C_{44}Mab-1 epitope. After antigen retrieval, sections were incubated with C_{44}Mab-1 (1 μg/mL) or C_{44}Mab-1 (1 μg/mL) plus human CD44 peptide (aa 471–490, 10 μg/mL). (B) The oral SCC tissue section was incubated with an isotype control mAb, PMab-44 (1 μg/mL). (C) A negative control tissue section (cat rectum) was incubated with C_{44}Mab-1 (1 μg/mL). The tissues were further treated with the Envision+ kit. The color was developed using DAB, and sections were counterstained with hematoxylin. Scale bar = 100 μm.
